# Editorial

**DOI:** 10.5152/TJG.2023.96123

**Published:** 2023-03-01

**Authors:** Filiz Akyüz

**Affiliations:** Department of Gastroenterology, İstanbul University İstanbul Faculty of Medicine, İstanbul, Turkey

Capsule endoscopy (CE), which has been in the practice of gastroenterology for more than 20 years and is one of the basic imaging methods in the visualization of the small intestine lumen, is becoming increasingly popular. The small intestine, which is the longest part of the gastrointestinal tract and is the most difficult to reach anatomically with conventional endoscopic methods, can be made visible by capsule endoscopy. Its non-invasive feature, providing patient comfort, and easy applicability increase its preference in practical application for both the patient and the doctor. Although it is one of the basic methods for imaging the small intestine lumen, capsule types have also been developed that enable imaging of the esophagus and colon. Capsule endoscopy, which is one of the areas where the reflection of technological developments is seen mostly, will be applied in the future not only for diagnostic but also for therapeutic procedures.

Although the applicability of capsule endoscopy is easy, there are rules to be considered in details in daily practice. This article tried to present the process from the preparation of the patients before the capsule endoscopy to the writing of the capsule endoscopy reports, with the aim of guiding the gastroenterologists who perform the capsule endoscopy.^1^

While all international guidelines are basically similar, they may have some differences. Last European Society of Gastrointestinal Endoscopy (ESGE) does not recommend routine second-look endoscopy prior to small-bowel capsule endoscopy in patients with suspected small-bowel bleeding or iron-deficiency anemia. This recommendation is strong, but low-quality evidence.^2^ However, in practice, gastroscopy and colonoscopy should be repeated, if duodenum third/fourth part and ileum are not examined. Therefore, this article recommends a second look if endoscopic examination is incomplete. This consensus reported that main indications of CE are OGIB (overt/occult gastrointestinal bleeding) and IDA (iron deficiency anemia).^1^ Small bowel obstruction is the only accepted contraindication for CE currently. But we do not recommend routine small bowel imaging studies for capsule retention before CE. On the other hand, swallowing disorders and dysmotility of GI system should be questioned carefully. Bowel preparation is important before CE for perfect visualization so that we recommend bowel preparation before CE.

This concensus recommend ileocolonoscopy and imaging study before CE in patients with suspected Crohn’s disease (CD). In the patient without obstructive symptoms, CE may be the first choice after at least intestinal ultrasound examination has confirmed that there is no obstruction.

Detailed and clear documentation is very important for the management of patients. Balloon enteroscopy can be used as a complementary endoscopic procedure to CE, especially for therapeutic purposes. The choice should be determined according to patients’ clinical characteristics in our daily practice.

As a conclusion, this consensus tried to present the details of the use of CE in practical medicine, taking into account international guidelines, as a recommendation based on expert opinion.
